# Wonder, delusions and meaning

**DOI:** 10.1192/bjb.2021.80

**Published:** 2022-08

**Authors:** Christopher Donald Baldwin

**Affiliations:** Denbigh, UK

**Keywords:** Philosophy, intentionality, psychosis, conscious awareness, delusions

## Abstract

Strands of thought in the philosophy of mind offer another way of looking at the nature of mental illness and how it arises from intense emotional states. Analysing the phenomenon of wonder is suggested as a novel approach to explaining delusions and variations in insight.

A recent Against the Stream article in this journal asking why neuroscience hasn't delivered for psychiatry emphasised that the complexities of human pathological emotional reactions cannot obviously be attributed to biological malfunctions.^1^ More investigation of the psychosocial origins of mental illness were suggested as a better way forward in understanding and treating severe illness. I suggest that, as well as biology and psychosocial factors, there is another angle which is due for more consideration.

Almost 30 years ago K.W.M. Fulford remarked that the mind–brain debate in philosophy and the nature of mental illness debate in psychiatry ought to feed off each other, and he suggested that analysing the concept of action was a way forward.^2^ He pointed to the philosophical work of John Searle on intentionality,^3^ in which the way that people relate to things in the world (whether physical items, social situations or novel ideas) depends on their background experiences and understandings. We all rely on our ‘background’ to tell us what to do in familiar situations without having to consciously think about it. This is our ‘know how’ not our ‘know what’. Our beliefs and attitudes, including confidence in our abilities, which inform all our concepts about the world, are built up from perceptions gained from life experiences to form what Searle calls ‘the Network’.^4^

Research in the reductionist tradition tries to link mentality to specific brain activity and so give a neurological explanation for all aspects of mind. The ‘eliminativist’ project championed by philosophers Paul and Patricia Churchland would have us abandon the term ‘belief’ in favour of descriptions about which particular brain cells depolarised, and in what sequence they did so, to give recognition of a pattern reflecting something about the real world.^5^ No duality of mind and brain is required in this scheme because everything supposedly mental can be explained by material cause and effect in the brain. An example of such an approach utilising the phenomenology of wonder is the electroencephalogram recordings of brain states in astronauts experiencing spectacular views of the earth from space.^6^ In terms of understanding the psychiatric states in which delusional ideas loom large, I contend that, necessary though those brain events are to allow mental phenomena to occur, a more subtle appreciation is required than just measuring physical events in particular areas of the brain.

## The importance of wonder

Wonder is a conscious state familiar to us all. The ‘wow’ moment, even when seen digitally as a 2-D face with open mouth and raised eyebrows, provides recognition of an unmistakable feeling. That feeling or emotion seems to be the trigger for action both to determine the cause of the wondrous event and to think about its nature and consequences. Plato said ‘This sense of wonder is the mark of the philosopher. Philosophy indeed has no other origin’.^7^ Aristotle considered that the experience of wonder (ancient Greek, *thaumazein*) was the driving force behind scientific endeavour in order to produce an understanding of the ways in which nature worked.^8^ In more recent philosophy there has been a concern that an overemphasis on scientific explanations diminishes the degree of wonder experienced, because if the mechanisms are commonly known there is less reason to feel in awe of those particular phenomena. Our modern feelings about lightning strikes and eclipses are two classic examples: less awe and dread, more of a passing interest and recognition. Twentieth-century philosophers worried about this. Wittgenstein was especially critical about a culture that concerned itself with causes while downplaying meaning^9^ and Howard Parsons talked of it extinguishing from awareness the qualitative uniqueness of things.^10^ Lately, environmental concerns have raised awareness that for the sake of Nature itself there is indeed a cultural problem with reductive explanations.^11^

Psychiatrists are interested in how strange ideas arise, what meaning they carry for the patient and if they are likely to lead to behavioural abnormalities. A wonderful experience might remain in the memory as just that, but if it leads to unusual interpretations that become dangerous to the patient or others, psychiatrists are usually asked to supply a third-party understanding of why this has taken place. Organic brain disease is an obvious worry and cognitive tests are available to check (at the very least) orientation, ability to concentrate and aspects of the patient's memory. This is the biological approach to investigating ‘cognition’ in the sense of ability to think. In addition, a psychosocial approach might emphasise educational or cultural reasons for the patient's behaviour. Both those approaches are undoubtedly valid but there seems merit in trying to understand how the experience of a subjectively dramatic event (the ‘phenomenology’ in Edmund Husserl's sense of ‘going back to the things themselves’)^12^ can induce what appears to be (to the rest of us) a state of twisted logic, often accompanied by signs of heightened physiological arousal and declarations of fear, anger or distress.

## Subjective significance

In the presence of something causing extreme astonishment, marvel, dread or awe, which historically constituted different variants of wonder,^13^ one inescapably knows that something dramatic is taking place. In Wittgenstein's words, human beings can ordinarily see and hear and feel as a matter of general experience and ‘So they are their own witnesses that they have *consciousness*’ (his italics).^14^ With consciousness comes subjective significance and a ‘what it is like’ to have a specific awareness. An awareness of something seems to be an overall feeling as well as an intellectual appreciation, not simply the registration of a cognitive pattern. Yes, flashes of colour can be recognised and buttons pressed to determine P300 intervals (the neuropsychology of which is discussed by Polich^15^) but what does that feel like – and why might it matter? Notions about inhibition of gated ion channels in neuronal circuits are not easily reconciled with wondering about significance and purpose.

Psychiatrists are familiar with the behavioural consequences of delusional ideas experienced by patients. Combatting a person's terror about what seems to be happening is of course a major concern but extreme fear is not the only emotion produced by delusional states. Puzzlement and consternation are often evident, particularly in both the early and the resolving stages. Trying to understand the mental mechanisms producing the strange beliefs is one thing but why there might be glimmerings of insight is another important therapeutic consideration. In the literature concerning the evaluation of insight there have been attempts to quantify its presence or absence with questionnaires such as the Beck Cognitive Insight Scale,^16^ which pays particular attention to how well individuals view their ability to reflect on their judgements (self-reflectivity) and how certain they are about those decisions (self-certainty). Although developed for use with people with psychosis, some validity has been claimed for its use with non-psychotic people.^17^ The essential point about this endeavour is that it is a purely cognitive one (assessing the ability to think), with little regard paid to the importance of emotion or feeling. There is actually one question in the ‘self-certainty’ scale that asks for agreement or otherwise on the statement ‘If something feels right it means that it is right’. Intuitively, a psychiatrist would probably guess that someone experiencing psychosis is more likely than a non-psychotic person to agree with such a sentiment. Wittgenstein said that in questioning the truth of a situation one must remember: ‘from its *seeming* to me – or to everyone – to be so, it doesn't follow that it *is* so (his italics). What we can ask is whether it can make sense to doubt it’.^18^ I think that the failure of the ability to doubt something which suddenly just feels right must be accounted a psychotic symptom and illustrates the importance of paying attention to how the patient experiences a significant event.

## Thought and feeling in conflict

Rationality relies on intact cognitive capacities to reject outlandish ideas, so at some point in the formation of a delusion there must be a breakdown or an overwhelming of that function. Established delusions are by definition an intellectual fixture but in their development the patient often exhibits great puzzlement, or perhaps uncertainty, at contradictions that are still apparent in his or her thoughts. The same upset can accompany residual or returning insight. It seems to me that there is something akin to a debate going on in the patient's mind, a debate concerning the truth or falsehood of previous unquestioned assumptions versus a possible new understanding. The problem is that ‘emotion’ (etymologically, something out of which activity occurs) accompanying those ideas can drive matters on towards a frenzy.

The philosophical analysis of wonder, as an experiential state in non-psychotic people, links a definite happening in the outside world with an overwhelming feeling that forces complete attention on the remarkable situation.^19^ An emotion may be defined as ‘an occurrent conscious state, with a certain affect, and with a certain kind of intentional content’.^20^ No emotion other than wonder has such a clear relationship with the outside world, being directly produced by the wondrous event. Something happens, triggering an emotion (Wow!), accompanied by a realisation that things do not quite fit together anymore, in that a previous belief about what was possible or true suddenly seems in doubt. This is intellectually astonishing and demands thought over time in the aftermath of the initial shock, producing ideas about what it is that has happened and what it might mean; and feelings about that meaning may seem to require action. This may be rational action in terms of a scientific endeavour to clarify the cause of the wonder, as in Aristotelian *thaumazein* (which would, strictly speaking, also ask questions about purpose), or perhaps lead to just talking loudly about it in an erratic fashion. Some might indulge in outright conspiracy theorising, depending on personality and their previous network of beliefs. Unfortunately, in the presence of severe mental illness actions felt to be subjectively necessary may turn out to be completely irrational and dangerous.

Descartes commented that emotions such as joy and love are different from wonder because they rely first on an internal decision by the intellect whereas wonder is a feeling triggered by something external.^21,22^ Wonder at an internally generated idea, perhaps such as a mathematical formula, must be possible but using the terms ‘inner’ and ‘outer’ about the mind do represent an outdated Cartesian duality. As perceptively observed by Hao Tang: ‘Our sensations, insofar as we are rational animals, are already infused with conceptual content, already shaped by the hand of reason’.^23^ Stephen Mulhall goes further than just sensation with his take on being in the world: ‘we encounter the world as always already saturated with human *meaning*’ (my italics).^24^ So in the process of recognising wonder, the initial ‘jangling of brain cells’ is followed by mental turmoil trying to make sense of the astonishing event and fit it into some sort of consistency with the concepts about the world contained by an individual's network. No doubt there are several neural correlates underlying all this but at a metaphysically higher level (of consciousness) one can view an individual's sense of wonder as a cognitive recognition of the implication that relationships between concepts have been broken plus a radical astonishment that this could have happened. This recognition stimulates the feeling that may best be described simply as ‘Wow!’ or as something more lyrical, such as the poetic rendition of an epiphany (examples of such poetry are given by Chappell^25^).

## Doubt overwhelmed by emotion

Autochthonous (primary) delusions, in which new meaning spontaneously arises about some previous understanding,^26^ seem to me to have the same trigger factor(s) as novel ideas. An observation invalidates a previous belief or hypothesis. Something apparently outlandish and incomprehensible has occurred. How can the implications be reconciled with the rest of the network? Remembering that Aristotle's appreciation of *thaumazein* contained a search for purpose as well as material cause, our modern intellectual approach looking for that cause may be missing an important interpretive angle. I suggest that although curiosity about truth is usually acceptable as a motive force, an emotional desire to find a particular interpretation that perhaps carries an ideological slant begins to look dubious. In terms of modern scientific endeavour, seeking after truth is generally considered as the paradigm of rationality – and being influenced by emotion is erratic. The switch to what we recognise as a psychotic state occurs at some point a little further along the scale of emotional balance. Then the normal cognitive correction (the ability to doubt) to emotional shock fails and the suddenly discovered meaning, however strange and dreadful, has to be seriously entertained by the individual under pressure from the tidal wave of the feeling that constitutes radical astonishment. The implications of any conclusions may themselves instigate further emotions, such as awe and dread (linguistic siblings of wonder) but more especially fear. In the presence of a developing psychosis an intellectual and emotional struggle has to take place, which all psychiatrists have observed, in what is usually described as a tortured mind.

How satisfactory or upsetting the patient finds the result of the battle is, I suggest, the key to an observer understanding the behavioural consequences of the illness. Will it settle into a set of understandings that are compatible with relatively normal social life, or will there be actively antisocial sequelae? Concentrating on possible neuropathology is vitally important because abnormalities clearly impinge on memory, beliefs, desires and intellectual abilities. Are neurological problems sufficient explanation for the radical misinterpretations (assuming they are indeed completely irrational) made by people with psychosis? It is possible to imagine minimal pathology and yet a sufficiently impaired overall awareness so that once a psychotic illness has taken hold the network is corrupted and all sorts of interrelationships between concepts are disrupted. The initial point of disruption is when the delusional idea is developing owing to the wondrous experience, which is not only puzzling and perhaps frightening but taken as increasingly convincing evidence that old ideas are unworkable. Similarly, on recovery, as the delusion begins to disintegrate and the previous network of beliefs looks more and more plausible again, there is often the rejection of any suggestion that the psychotic experience ever happened, which is a more welcome behavioural response.

## Conclusion

Fulford commented that ‘the relationship between normal belief and action is tricky enough to disentangle’, so understanding psychotic actions is bound to be more difficult.^27^ Applying Searle's intentionality to the phenomenology of wonder is a philosophical task involving the analysis of mental mechanisms and conscious states leading to questions about meaning and purpose. This sheds light on how the mind might actually work. Psychiatric experience shows that, in forming understandings about objects in the world, much may go awry and then the abnormal can be used to illuminate the normal. However, my suggestion here is that the normal mental turmoil of wonder in which one's usual concepts are challenged provides a way into seeing how the process of forming a delusion might arise and how problematic behaviours might result. Philosophy thus illuminates psychiatry and, in return, the encounter with psychotic activity reminds philosophers of the reality of severe mental illness.

## About the author

**Christopher Donald Baldwin**, MBBS, BA, MA, MRes, MRCPsych, DGM(RCP), is a retired consultant psychiatrist in North Wales, UK.
